# Schizofrenia: Study of Tendency and Distribution

**DOI:** 10.1192/j.eurpsy.2022.358

**Published:** 2022-09-01

**Authors:** C. Vilella Martín, P. García Vázquez, P. Fernández Perea, Y. Barrera García, A. Serrano García, R. Gómez Martínez, C. Franch Pato

**Affiliations:** Complejo Asistencial Universitario de León, Psychiatry, LEÓN, Spain

**Keywords:** schizophrénia, distribution, Hospitalizations

## Abstract

**Introduction:**

The prevalence of schizophrenia is close to 1 percent internationally. According to the 2019 census, the population in the province of León, our study population, is 460,001 inhabitants.

**Objectives:**

To study the distribution of schizofrenia in the area covered by the Complejo Asistencial Universitario de León, Spain.

**Methods:**

This is a retrospective and cross-sectional descriptive study. The data of the hospitalizations of the last 10 years (2009-2019) will be obtained in any service of the CAULE of the 28 basic health areas of the province of León, with a diagnosis of schizophrenia. Prevalence will be calculated. The rate of schizophrenia will be calculated for the decade per 1000 inhabitants.

**Results:**

3133 admissions identified 1576 unique patients. It is the decade of 50-59 where the largest number of hospitalizations is concentrated. Most entered directly into the psychiatry hospital care. It is 2019 where the most income is produced and 2017 the one with the least. The rate of schizophrenia is 3,2 Per 1000 inhabitants.

**Conclusions:**

Hospitalizations for schizophrenia is concentrated in the decade of the 40-49 years. The diagnosis of schizophrenia is frecuently delayed until negative symptoms appear. There is an upward trend in hospitalizations per year in the last decade. The rate of schizophrenia is higher in areas where consanguinity is present and where the prison is located.

**Disclosure:**

No significant relationships.

